# Early rise in nasal secretory IgA associated with shorter duration of SARS-CoV-2 virus shedding in an acute infection cohort

**DOI:** 10.3389/fimmu.2026.1722585

**Published:** 2026-02-25

**Authors:** Caroline Brackett, Lu Zhang, Bich T. N. Do, Saman Baral, Leigh H. Fisher, William O. Hahn, Alexander J. Carnacchi, Sir’Tauria Hilliard, Shuying S. Li, Lakshmanane Premkumar, Alexander L. Greninger, Ollivier Hyrien, Kelly E. Seaton, Georgia D. Tomaras

**Affiliations:** 1 Departments of Surgery and Integrative Immunobiology, Center for Human Systems Immunology, Duke University School of Medicine, Durham, NC, United States; 2 Vaccine and Infectious Disease Division, Fred Hutchinson Cancer Research Center, Seattle, WA, United States; 3 Department of Microbiology and Immunology, University of North Carolina, Chapel Hill, NC, United States; 4 Department of Laboratory Medicine and Pathology, University of Washington Medical Center, Seattle, WA, United States

**Keywords:** mucosal IgA responses, SARS-CoV-2, secretory IgA, serum IgA, viral shedding duration

## Abstract

**Introduction:**

Understanding the role of mucosal immune responses in preventing coronavirus replication is essential for the development of broad-spectrum therapeutics and vaccines capable of interrupting transmission. Investigating the relationship between mucosal antibodies and viral shedding may provide critical insights into protective mechanisms against SARS-CoV-2 and inform strategies for enhancing mucosal immunity.

**Methods:**

In a subset of the larger CoVPN 5001 cohort, 143 participants from the US, Mexico, and South America were characterized as either short (<7 days), intermediate (7–21 days), or prolonged (>21 days) virus shedders based on RT-qPCR measurements over the first month of acute SARS-CoV-2 infection. We measured systemic circulating serum IgA and secretory IgA (SIgA) in serially collected nasal lavage fluids to 15 SARS-CoV-2 antigens, including spike variants, receptor binding domain (RBD), and N-terminal Domain (NTD) and evaluated the relationship between antibody titers and duration of viral shedding using multivariate and binary logistic regression modeling. We also assessed antibody responses to RBD proteins of endemic alpha and beta coronaviruses to compare variations in antibody kinetics throughout the course of infection.

**Results:**

We found that increased serum IgA and mucosal secretory IgA antibody titers during the earliest phase of acute infection were associated with decreased viral shedding duration, with nasal IgA responses to the spike NTD being the strongest factor associated with viral shedding (adjusted p<0.1). In contrast, antibody titers against endemic human coronaviruses remained stable throughout the course of infection, consistent with a specific role for newly elicited SARS-CoV-2 antibodies in virus control.

**Discussion:**

These findings demonstrate that early rises in serum and mucosal IgA titers are associated with reduced duration of viral shedding. Notably, nasal secretory IgA responses targeting the spike N-terminal domain were most strongly associated with shorter shedding, suggesting a potentially protective role for SARS-CoV-2-specific nasal IgA during acute SARS-CoV-2 infection. These results highlight the importance of mucosal immunity for limiting viral transmission and shedding, offering future insights for pan-coronavirus therapeutics and vaccine development aimed for enhancing SARS-COV-2 mucosal antibody responses.

## Introduction

SARS-CoV-2, the virus that causes COVID-19, is now endemic, with at least 775 million cases reported worldwide since 2019 (1). Vaccinations and natural immunity have contributed to the decrease in disease severity and hospitalization, but significant morbidity is still associated with clinical SARS-CoV-2 infections (2–4); an efficacious pan-coronavirus vaccine may pave the way for better protection against current and future viral strains (5–8). An improved understanding of coordinated innate and humoral responses at the mucosa, stimulated by upper respiratory viral infections such as COVID-19, Influenza, and RSV (9), is needed for design of new therapeutic and preventative strategies.

Natural control of virus replication in acute SARS-CoV-2 infection varies among individuals and contributes to differences in symptoms and disease severity (10–12). Secretory IgA (SIgA) responses are a major component of the mucosal immune response against respiratory pathogens, and prior research has identified a critical role for serum IgA to neutralize SARS-CoV-2 during the early stages of infection (13). Individuals deficient in IgA fail to mount an adequate humoral response to either infection or vaccination, indicating that antigen-specific IgA neutralization may influence disease severity, vaccine efficacy, and viral shedding (14). The nasal compartment, a vulnerable entry point for SARS-CoV-2 transmission via aerosols and droplets, features mucosa in the respiratory tract that serves as the first line of immune defense against viruses (15). SIgA, a complex of dimeric IgA and secretory component (SC), is present in secretions in the upper respiratory tract and functions to neutralize pathogens by aggregating immune complexes, thus preventing further entry into the tissue and the establishment of infection (13, 16). The natural kinetics and specificity of SIgA in nasal fluids during acute SARS-CoV-2 infection are unknown. Thus, we explored IgA antibody roles in early infection, in addition to evaluation of secretory IgA responses in the upper respiratory mucosa.

This study utilized an international longitudinal cohort of naturally infected, non-vaccinated individuals to better understand the immune responses that impact the duration of SARS-CoV-2 viral shedding. We hypothesized that the presence and magnitude of early nasal secretory antibody responses to SARS-CoV-2 would decrease the duration of viral shedding in the upper respiratory tract.

## Results

### Acute SARS-CoV-2 longitudinal infection cohort

Participants in the CoVPN 5001 cohort were enrolled from North America, South America, and Mexico; samples were collected between July 2020 and August 2021 (24). At enrollment (visit 1), participants were positive for SARS-CoV-2, by RT-PCR or antigen testing, and samples were collected for a total of 5 visits over the course of 28 (± median=7) days. In the larger CoVPN 5001 cohort, viral shedding duration was categorized using the following categorization: short shedding (<=7days); intermediate shedding (7-21days); and prolonged shedding (>21days). A subset of participants was selected on the basis of belonging to one of the three shedding categories and matched on demographic variables including age, sex, region, and comorbidities (percentages shown in **Table 1** for each group). Serum and nasal washes collected from each visit were assessed for IgA and secretory IgA (SIgA), respectively, where antibody binding responses were measured against both SARS-CoV-2 and endemic Human Coronavirus (HCoV) proteins.

**Table 1:** Acute SARS-CoV-2 infection cohort information.

Demographic categories	SARS-CoV-2 shedding duration			Total
	<=7 days	(7,21] days	>21 days	
N	23	66	54	143
Region				
Mexico & South America	17 (73.9%)	29 (43.9%)	32 (59.3%)	78 (54.5%)
United States	6 (26.1%)	37 (56.1%)	22 (40.7%)	65 (45.5%)
Sex				
Female	9 (39.1%)	30 (45.5%)	24 (44.4%)	63 (44.1%)
Male	14 (60.9%)	36 (54.5%)	30 (55.6%)	80 (55.9%)
Age				
Mean (SD)	38.7 (10.9)	40.7 (11.8)	38.9 (14.6)	39.7 (12.7)
Median	38.0	40.0	36.0	39.0
Range	19.0 - 67.0	18.0 - 71.0	20.0 - 70.0	18.0 - 71.0
IQR	32.5 - 46.0	34.2 - 48.8	26.0 - 47.2	30.0 - 47.5
BMI				
Mean (SD)	27.8 (4.4)	28.2 (5.1)	27.1 (4.2)	27.7 (4.7)
Median	26.6	27.0	27.0	27.0
Range	20.7 - 36.6	19.0 - 40.7	19.4 - 38.2	19.0 - 40.7
IQR	25.5 - 30.1	24.4 - 31.2	24.0 - 30.2	24.4 - 30.7
HIV				
No	21 (91.3%)	51 (77.3%)	41 (75.9%)	113 (79.0%)
Yes	2 (8.7%)	15 (22.7%)	13 (24.1%)	30 (21.0%)
Hypertension				
No	20 (87.0%)	57 (86.4%)	46 (85.2%)	123 (86.0%)
Yes	3 (13.0%)	9 (13.6%)	8 (14.8%)	20 (14.0%)
Current Smoker				
No	17 (73.9%)	50 (75.8%)	44 (81.5%)	111 (77.6%)
Yes	6 (26.1%)	16 (24.2%)	10 (18.5%)	32 (22.4%)
Asymptomatic				
No	18 (78.3%)	61 (92.4%)	53 (98.1%)	132 (92.3%)
Yes	5 (21.7%)	5 (7.6%)	1 (1.9%)	11 (7.7%)
Covid days at enrollment				
Mean (SD)	5.0 (1.6)	8.3 (3.5)	8.2 (3.4)	7.8 (3.4)
Median	5.0	9.0	8.0	7.0
Range	2.0 - 7.0	2.0 - 15.0	2.0 - 17.0	2.0 - 17.0
IQR	3.5 - 6.0	5.0 - 11.0	6.0 - 11.0	5.0 - 10.0

### Secretory IgA is abundant in nasal washes

**Figure 1:**
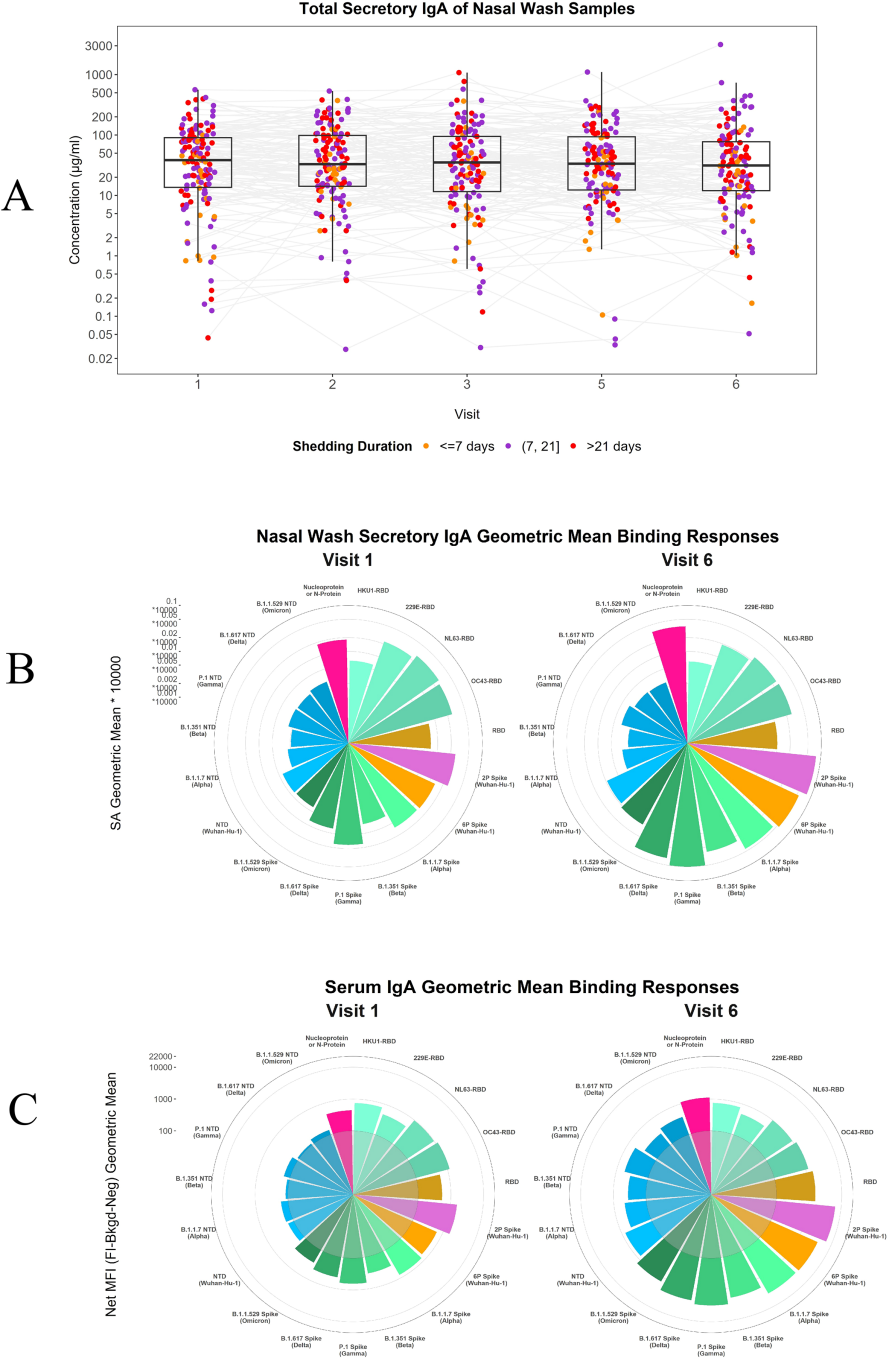
Total SIgA concentrations collected in nasal wash samples and breadth of SARS-CoV-2/Endemic HCoV antibody responses over time. Total secretory IgA concentrations and antigen specific secretory IgA (Nasal)/serum IgA antibody responses throughout acute SARS-CoV-2 infection: **(A)** Total SIgA concentrations in µg/ml from day of COVID onset (Visit 1) throughout acute infection (Visit 6) for short (<=7days), medium ((7,21] days), and long (>21 days) viral shedders; median, inter and outer quartile ranges shown for each visit. **(B, C)** Geometric mean of binding antibody antigen specific responses for nasal washes (secretory IgA) and serum (IgA) at visit 1 and visit 6. Inner gray circle represents 100 net MFI range on **Figure 1C**.

Nasal washes were collected and aliquoted at clinical sites and total SIgA was measured in nasal specimens via Binding Antibody Multiplex Assay (BAMA). SIgA was detected at levels >0.25 µg/ml in 96% of samples tested across all time points (Median = 36.12 µg/ml; Range [0.25 – 3145.59 µg/ml]). A majority of these nasal wash collections exhibited high levels of total SIgA, indicating the presence of antibody collected for this sample type. (**Figure 1A**; **Supplementary Table 2**). P-values for the unique group comparisons were corrected for multiple testing using the FDR method. All adjusted p-values were >0.2 after multiple testing correction, indicating no statistically significant differences between groups at any timepoint.

### Antigen specific breadth of responses over time

Following total SIgA measurements, antigen-specific responses were measured in the mucosa (SIgA) and serum (IgA), with matched specimen types for the same participant. Secretory IgA and serum IgA antibody levels show a marked increase in anti-SARS-CoV-2-specific responses from the onset of COVID-19 to the final visit time point, specifically for spike (Wuhan-1 and variants) and the NTD Wuhan-1. (**Figures 1B, C**; **Supplementary Table 3**). This highlights the progression and augmentation of antigen-specific antibodies over the course of the infection.

The relationship of SARS-CoV-2 specific serum IgA and nasal SIgA were next compared over time, to investigate variations in the timing of antibody responses. McNemar Test was utilized to compare the overall response rates between the first and last visits. Significant differences were found for the spike proteins (Wuhan-1 and alpha, beta, gamma, delta, and omicron variants; FDR adjusted p values <0.0001****) (**Supplementary Figure 1**).

### Persistent viral shedders exhibit delayed SARS-CoV-2 but not endemic HCoV responses in nasal washes (SIgA) and serum (IgA)

**Figure 2:**
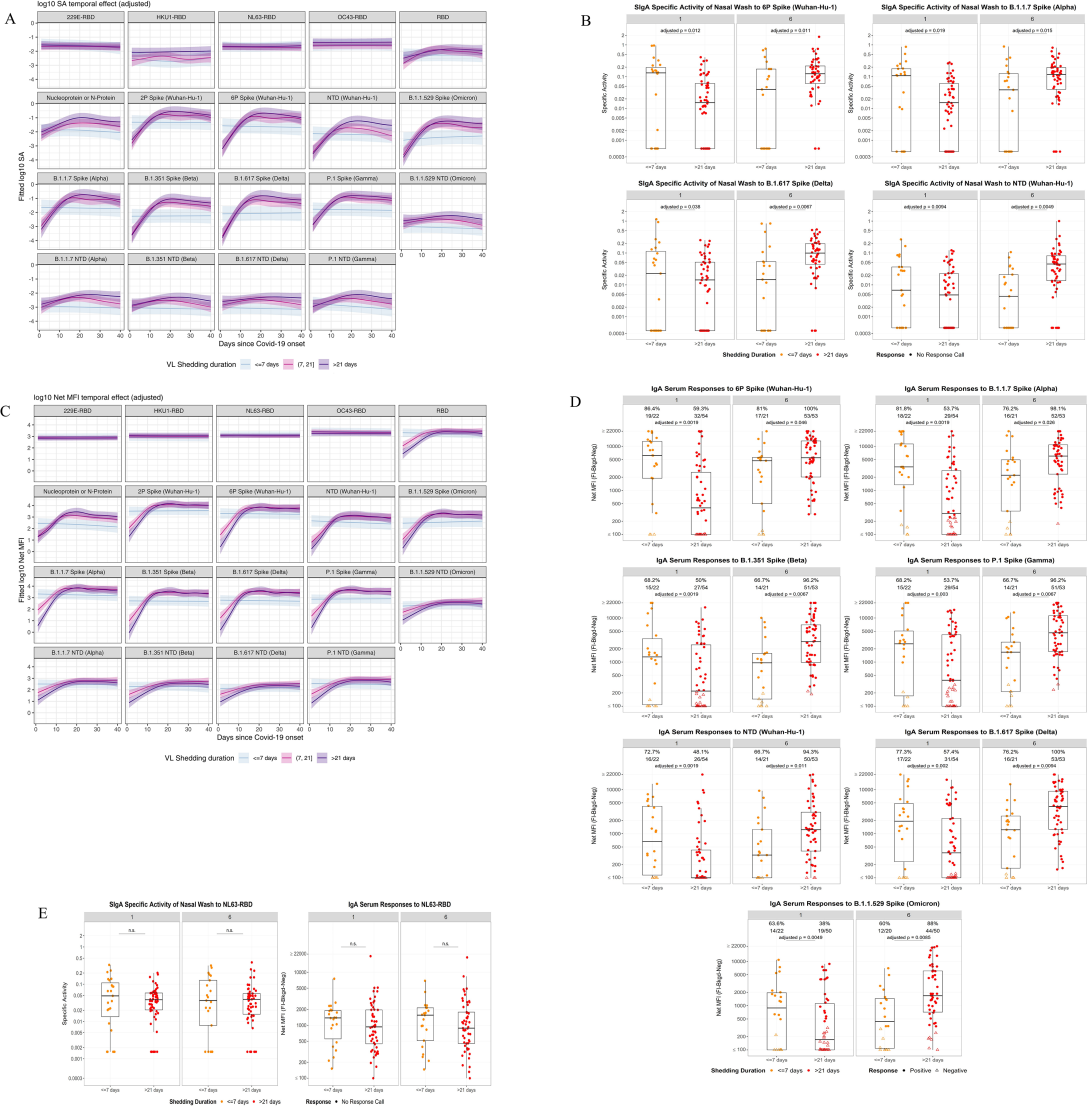
Mucosal and serum antibody kinetics over time, by viral shedding status. **(A, C)** Mucosal and serum antibody titer kinetics: Mucosal antibody responses were measured as a function of antigen specific activity and serum antibody responses were measured as a function of mean fluorescence intensity. Participants are separated based on viral shedding category (<7 days, (7-21] days, and >21 days). Responses were measured against SARS-CoV-2 and endemic human coronavirus antigens (HCoV). **(B, D)** Mucosal and serum antibody responses for SARS-CoV-2/HCoV antigens at enrollment visit (visit 1: PCR+ test or symptom onset, whichever was first occurring) and final visit during infection time course (visit 6: 28 days post enrollment), evaluating short versus long shedding groups at each visit: Antigen specific immune response measurements were compared between short and long shedding categories at enrollment and final visit. Significant p-values are observed in antigen responses for both mucosal (specific activity on the y-axis) and serum (net MFI on the y-axis) antibody measurements, with visit 1 and visit 6 side by side for each antigen. **(E)** Antibody responses to the endemic human coronavirus NL63 were not associated with viral shedding duration, the analysis yielded non-significant p-values.

To further examine dynamic antigenic antibody responses within this cohort, we evaluated mucosal (**Figure 2A**) and serum (**Figure 2C**) antibody kinetics stratified by viral shedding status. Significant differences were observed between the short and long shedding group for nasal SIgA SARS-CoV-2 antibody kinetics at the first and last visits [FDR adjusted p<0.1 for 4 antigens at visit 1 and 6] (**Figure 2B**). Participants with short shedding had increased nasal IgA at early timepoints (visits 1 and 2) but ultimately had lower levels of nasal IgA beginning around 7–10 days after the onset of symptoms. This pattern is similarly evident among IgA antibodies in serum [FDR adjusted p<0.1 for 7 antigens between both visits] (**Figure 2D**). Significant antibody response differences between short and long shedding categories were not observed for the endemic coronaviruses in both the serum and nasal IgA samples, emphasizing the specificity of SARS-CoV-2 antibody maturation throughout the infection time course (HCoV NL63 shown in **Figure 2E**). This evidence supports the hypothesis that variances in initial antibody titers, in relation to shedding categories, are specific to SARS-CoV-2 antigens.

### Factors associated with duration of viral shedding

**Figure 3:**
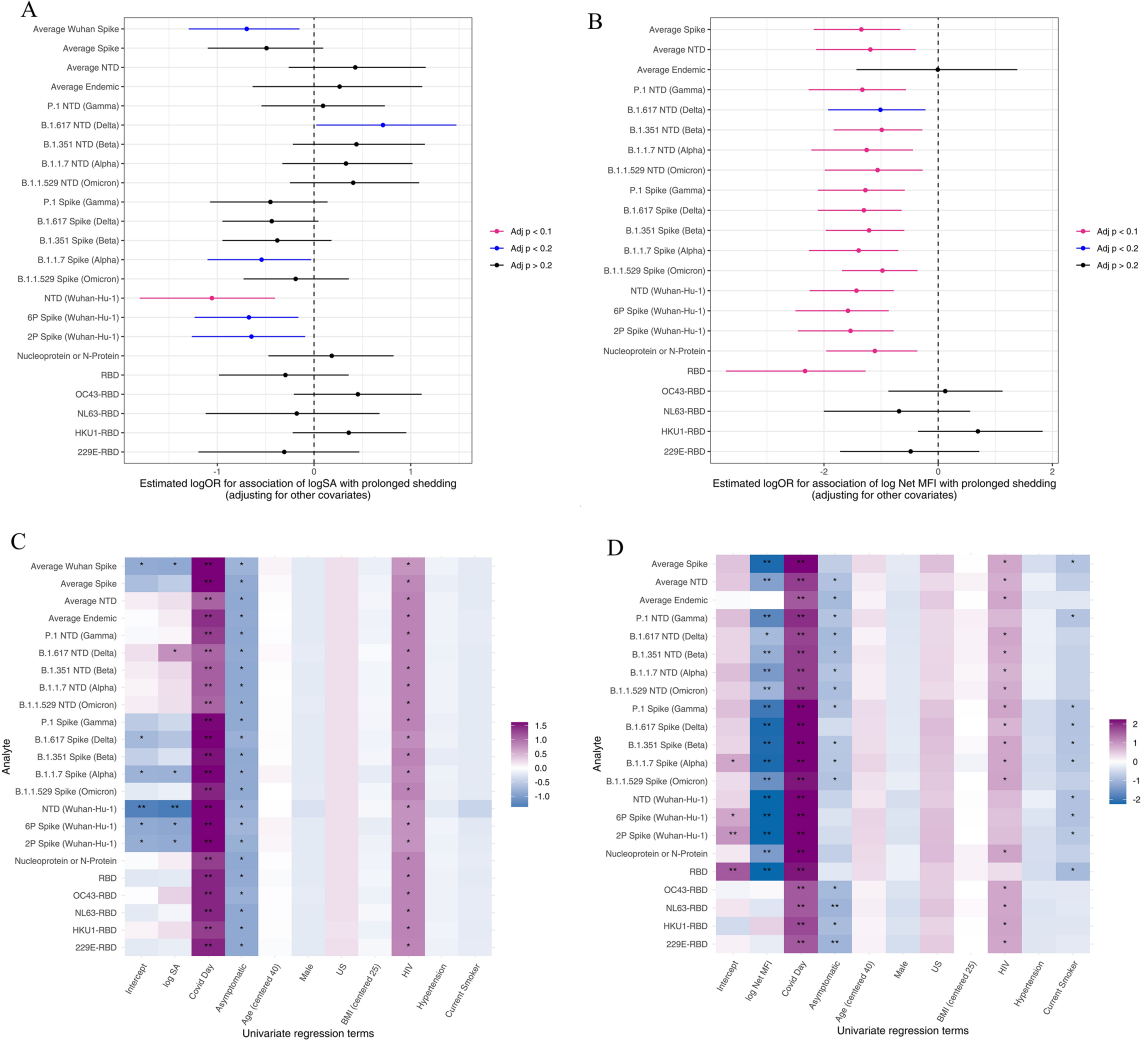
Factors associated with prolonged viral shedding as a function of binding antibody specific activity and MFI measurements. **(A, B)** Estimated logOR for association of biomarker responses to log SA **(A)** or log net MFI **(B)**, shown on the y-axis, with prolonged shedding. P<0.1 shows significant association with viral shedding, P<0.2 shows trending association with viral shedding; estimates represented by solid dots < 0 are associated with shorter viral shedding and > 0 are associated with prolonged viral shedding. **(C, D)** Univariate regression analysis prediction of viral shedding for association of baseline characteristics against biomarkers based on SIgA **(C)** or net MFI IgA **(D)** antibody responses. Heatmap colors indicate the signed log10 adjusted P-value, where dark purple indicates a significant positive association and dark blue indicates a negative association with prolonged shedding. **Adjusted p<0.1, *Adjusted p<0.2.

Using antibody titer responses for each analyte, a logistic regression model was used to determine the factors associated with shedding duration (<= 7 days vs. > 7 days) as a function of specific activity in nasal washes or Mean Fluorescence Intensity (MFI) in serum, at enrollment. For the mucosal antibody responses, significant associations (adjusted p-value <0.1) with lower shedding were found for higher anti-Wuhan-Hu-1 spike N-terminus domain (NTD) responses, while trending associations (adjusted p-value <=0.2) with lower shedding duration include higher anti-Wuhan-Hu-1 spike and anti-B.1.1.7 (alpha) spike; trending associations for longer shedding duration included higher anti-B.1.617 (delta) spike NTD responses (**Figure 3A**). For the serum antibody responses, significant associations with lower shedding duration include responses to spike, including NTD wild-type and variant strains, Nucleocapsid (N)-protein, and spike RBD with trending associations for higher anti-NTD (Delta) responses; no associations were significant for longer shedding duration by analyte (**Figure 3B**).

Prolonged viral shedding probabilities were also evaluated based on baseline characteristics. For both mucosal SIgA and serum IgA antibody responses at enrollment, longer shedding is associated with length of time since COVID-19 onset and HIV seropositive status for all biomarkers, while shorter viral shedding is associated with asymptomatic SARS-CoV-2 status (**Figures 3C, D**). Current smoking was found to be a significant association of shorter viral shedding for 11/22 biomarkers for serum IgA responses (**Figure 3D**). Viral shedding duration associations were not significant based on age, sex, region, BMI, or hypertension in either the mucosa or in serum.

## Discussion

In this study we identified that robust SIgA responses, along with serum IgA, are induced in early acute SARS-CoV-2 infection, and that persistent viral shedding for >21 days is associated with dampened initial serum and nasal responses. Humoral immune responses, whether mucosal or systemic IgA, exhibit significant distinctions across shedding groups, suggesting that early antibody defenses correlate with reduced viral shedding and potentially offer protection against prolonged disease progression. Additionally, shorter duration of virus shedding and viral load may minimize opportunities for virus mutation and transmission (17, 18).

Even though early systemic and mucosal responses are strong in the shorter viral shedding group, the correlation between SIgA and serum IgA are moderate to weak (R = 0.22 to 0.73) (**Supplementary Figure 2**). Similar findings were shown by Liew et al. where nasal SIgA and serum IgA levels were at best moderately correlated (19), likely indicating that natural infection priming of local respiratory lymph nodes play an important role in early clearance of the virus for some individuals. Additionally, vaccination with an mRNA booster vaccine markedly boosted SARS-CoV-2 specific IgA antibodies in nasal and salivary samples and may play a role in protection from virus acquisition or severe disease (20). This supports the concept of priming mucosal immunity, which has demonstrated protection against SARS-CoV-2 and influenza viruses, using a standalone intranasal immunization (9).

Since the samples measured in this study are limited to the acute phase of infection, we have not obtained data on the durability of natural infection or how viral shedding duration relates to protection against re-infection, highlighting one limitation of the study design. However, prior studies have suggested that systemic IgA in the sera are more durable than mucosal SIgA, especially 9 months post infection (19). Further exploration of the relationship between SARS-CoV-2 mucosal antibody levels and additional coordinated immune responses may be needed to understand protection against re-infection using longitudinal cohorts. Given the cooperative nature of the immune system, additional studies to examine the dynamics and levels of mucosal SIgA together with systemic immune responses will shed more light on the protective role of SIgA. We recently demonstrated in another cohort (where mucosal samples were not available) that coordinated systemic immune responses, comprising antibody and cellular responses, corresponded with a shorter duration of virus shedding (Schuster et al. Submitted 2025).

It is shown here that secretory IgA in the nasal compartment, induced by natural infection, elicits antibodies that target multiple spike epitopes (Receptor binding domain, N-terminal domain, and full-length spike). The epitope most strongly associated with viral clearance were spike-specific N-terminal domain (NTD) SIgA and receptor binding domain (RBD), serum IgA responses, suggesting that these epitopes are capable of mediating protection. Current licensed vaccines can provide some contribution to mucosal IgA responses, particularly in individuals with previous infection, however these responses are not long lasting, have minimal epitope targets, and require frequent repeated vaccinations (21). Our data demonstrates that an early rise in IgA is associated with a reduction in virus, providing rationale that vaccines eliciting IgA responses, targeting multiple epitopes, could block viral entry and disease progression. Intranasal SARS-CoV-2 vaccine design could additionally induce a more comprehensive systemic response, by mimicking the route of natural infection through the respiratory pathway (22), ultimately preventing infection and limiting viral transmission.

## Materials and methods

### Observational cohort, study design

In this prospective cohort study of acute immune responses to SARS-CoV-2 infection, 143 participants (11 asymptomatic; 132 symptomatic) from U.S., Mexico and S. America from a larger cohort were selected based on belonging to one of three viral shedding categories. Participants were enrolled based on presence of CoVID-19 symptoms or a positive RTQ-PCR test, whichever occurred first. As defined in the study protocol document, target visit window days with allowable widows are listed as such: Enrollment Target Day/Visit 1: 0 (-2 days), Visits 2 and 3: Target day 2, 7 (+/-1 day), Visits 5 and 6: Target day 21, 28 (+/-2 days).

The derived viral shedding categories were based on an individual’s ability to clear the virus (PCR confirmed) within 7 days (short shedding), between 7–21 days (intermediate shedding), and >21 days (prolonged shedding). See **Table 1** for demographic information (24). All participants provided informed consent prior to enrollment, and the protocol was approved by IRB protocol ID Pro00093087.

### Specimen collection and storage (at sites)

For nasal wash specimens, participants were given the option to self-collect or have clinical staff perform the collection procedure. 3-5mL of wash fluid (normal saline or PBS) was pipetted into one nostril, allowing the fluid to flow into the specimen cup. The process was repeated, alternating nostrils, until approximately 5-10mL of fluid was collected. Nasal wash specimens were sent to the processing lab (at 2-8 °C) and then frozen for specimen distribution. Blood was collected at clinical sites in SST tubes, and approximately 1mL serum was distributed to the lab.

### Binding antibody multiplex assay

SARS-CoV-2 specific IgA and secretory IgA binding antibody responses were measured against SARS-CoV-2 and endemic HCoV antigens on a Bio-Plex (Bio-Rad) instrument using a standardized custom Luminex based assay (23). SARS-CoV-2 and endemic HCoV specific mucosal antibody responses were measured at a 1:2 dilution and total mucosal secretory IgA measurements were measured at a 1:250 dilution. SARS-CoV-2 and endemic HCoV specific serum antibody responses were measured at 1:50 and 1:250 dilutions; concentration of total SIgA was calculated using a 5-PL logistic regression derived from a purified human SIgA standard curve. Briefly, biotinylated SARS-CoV-2 and endemic HCoV antigens were bound to NeutrAvidin coupled carboxylated fluorescent microspheres (MagPlex, Luminex). Antigen bound microspheres were then incubated with human sera or nasal wash, along with standards and controls, for 2 hours at 750 RPM and 22 °C. Mouse anti-human secretory component detection antibody (Sigma-Aldrich, clone GA-1) followed by Goat Anti-Mouse IgG PE (Biolegend, catalog 405307) were used to detect SIgA antibody responses in the mucosa. Goat anti-human IgA-PE (Jackson ImmunoResearch, catalog 109-006-011) was used to detect IgA serum responses. IgA serum samples were IgG-depleted before testing using a protein G MultiTrap plate (GE Healthcare Bio Sciences AB, Uppsala, Sweden). Assay plates were read on the Bio-Plex 200 (Bio-Rad) system providing a readout of background-subtracted MFI. Sixty-six SARS-CoV-2 seronegative serum samples (collected prior to November 2019, BioIVT) were tested at a 1:50 dilution and were used to establish antigen-specific positivity cutoffs for serum IgA. For mucosal samples, antigen specific secretory IgA antibody responses were normalized by dividing by total secretory IgA to account for differences in nasal wash collections. All samples, controls and standards were assayed in duplicate; negative controls and uncoupled microspheres were included in each assay to ensure binding specificity. Positive and negative controls were included in each assay to ensure assay consistency and reproducibility. Levey-Jennings charts were also utilized to track antigen performance throughout the study.

Anti-secretory IgA capture and detection antibodies were assessed for specificity to ensure secretory IgM antibodies were not being measured for antigen specific SIgA responses (**Supplementary Figure 3**).

These participants did not have pre-COVID-19 paired samples, therefore levels of pre-pandemic mucosal and serum cross reactivity with these antigens are not known. However, a separate set of pre-pandemic serum samples were tested against these antigens to determine non-specific background binding. This data was used to establish antigen-specific cutoff values for positive response calls.

### Statistical methods

First COVID Day was defined as either the day of earliest symptom onset or first positive PCR test, whichever occurred first. “COVID Days” was defined as number of days since the first COVID Day. Shedding duration was defined as COVID Days until viral clearance, defined as the first instance of two consecutive PCR-negative swabs, and categorized into three intervals: (0, 7], (7, 21], and >21 days. (24))).

Nasal wash SIgA specific activity (SA) was defined as the antigen-specific Net MFI divided by the Total IgA. As many of the antigens studied are highly correlated, we created the following summary measurements of the SIgA responses: Average Endemic response was defined as the geometric mean (GM) of the 4 endemic analytes; the Average NTD response was defined as the GM of the Alpha, Beta, Delta, Gamma, and Omicron NTD analytes; the Average Spike response was defined as the GM of the Alpha, Beta, Delta, Gamma, and Omicron Spike analytes. Similar measures were defined for the serum IgA measurements.

To study the kinetics of SIgA responses and the associated factors, linear mixed effects models were fit separately to the log specific activity of each analyte. As the primary interest was in the kinetics of the SIgA trajectories by viral shedding group, a generalized additive model (GAM) was fit with separate smooth functions of days since Covid-19 onset by viral shedding group. The model also adjusted for baseline characteristics, the baseline characteristics interaction with time, and individual-level random effects to account for the longitudinal data. The specific baseline characteristics included were: age at enrollment (centered at 40), sex assigned at birth, BMI (centered at 25), USA (yes/no), presence (yes/no) of human immunodeficiency virus (HIV), maximum disease severity (asymptomatic or not), hypertension (yes/no), and self-reported active smoker (yes/no).

Logistic regression was used to study the factors associated with prolonged viral shedding, defined as viral shedding greater than 7 days. For the analysis of each analyte separately, we fit logistic regression to model the probability of shedding >7 days as a function of log SA at enrollment, adjusting for time since Covid-19 onset (COVID days at enrollment), age (years, centered at 40), sex assigned at birth, region (USA or no), HIV status, maximum disease severity (asymptomatic or not), hypertension (yes/no), and current smoking status (yes/no). Similar models were fit using serum IgA responses in place of the SIgA response. P-values were adjusted for multiple comparisons by controlling the false discovery rate (FDR) using the Benjamini-Hochberg procedure. Differences were considered statistically significant when the FDR adjusted p-value were < 0.1 and trending for p-values < 0.2.

To determine whether antibody responses are associated with prolonged viral shedding (i.e., >21 days vs. <= 7 days) at early infection stage (enrollment visit) and final study visit, binary logistic regression models describing the probability of long shedding as a function of binding antibody titer were fit for each analyte separately. Each model adjusted for COVID days. Binding antibody titers (net MFI) for serum IgA and SA for nasal wash SIgA, were log-transformed and standardized prior to running the analysis.

McNemar test was used to assess changes in overall response rates throughout the infection, comparing the enrollment visit to the last visit. The overall response rate represents the proportion of participants who were positive serum IgA responders with nasal wash SIgA specific activity exceeding the threshold among all participants.

All hypothesis tests were two sided. P-values were adjusted for multiple comparisons by controlling the false discovery rate (FDR) using the Benjamini-Hochberg procedure. Differences were considered statistically significant when the FDR adjusted p-value was < 0.1 unless otherwise indicated. All statistical analyses were conducted using R version 4.1.3.

## Data Availability

Data can be requested from the HIV Vaccine Trials Network (HVTN) and Statistical Center for HIV/AIDS Research and Prevention (SCHARP). In keeping with NIH policies on data accessibility, we will make de-identified data publicly available on Atlas (a system designed to have PHI protections).
