# Supplementary figures and images for: Early rise in nasal secretory IgA associated with shorter duration of SARS-CoV-2 virus shedding in an acute infection cohort
Source: Front Immunol. 2026 Feb 25;17:1722585. doi: 10.3389/fimmu.2026.1722585 (PMC12975920; doi:10.3389/fimmu.2026.1722585)

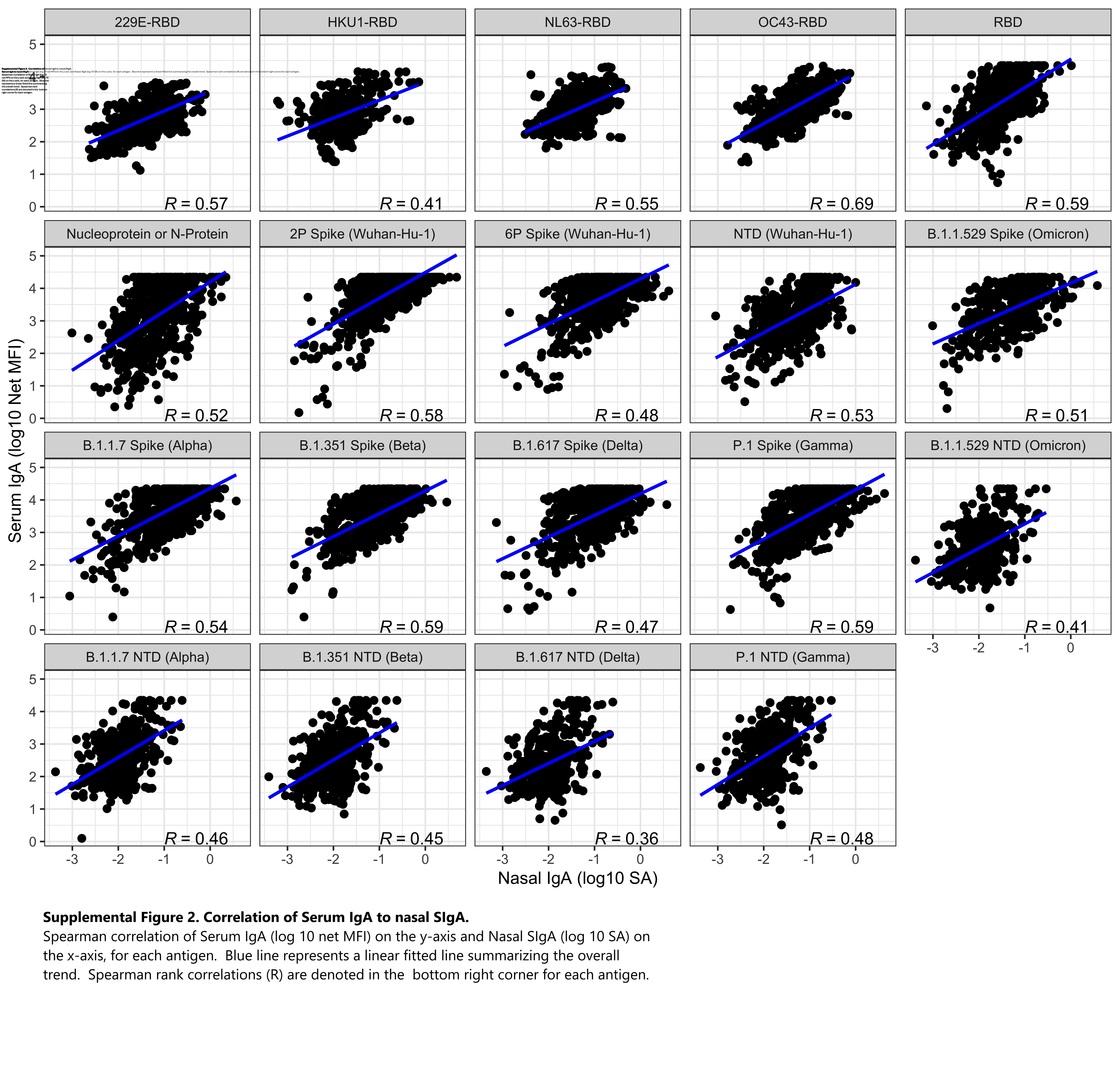

Supplement: Supplementary file 2 [file Image2.jpeg]

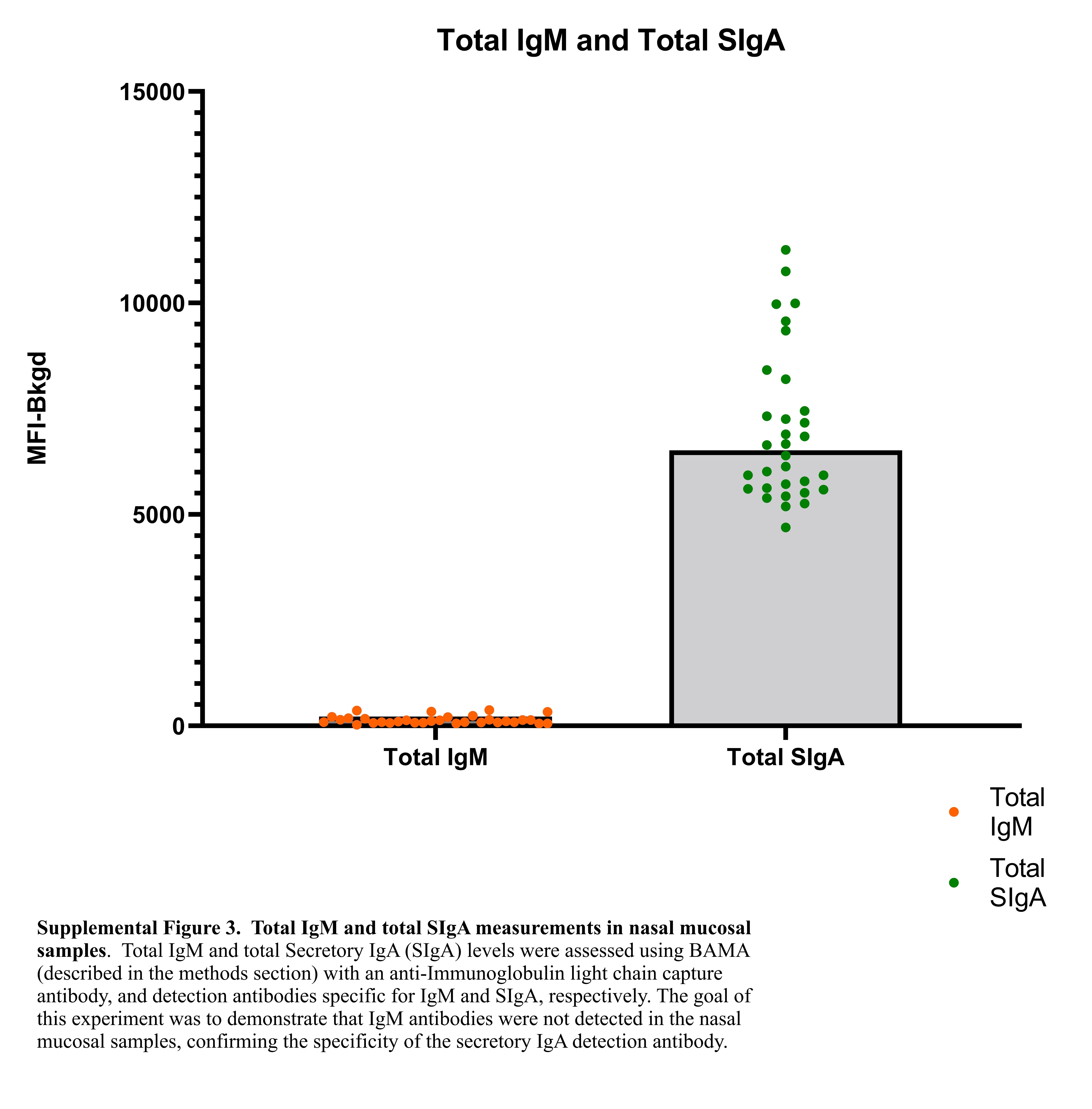

Supplement: Supplementary file 3 [file Image3.jpeg]
