# Supplementary material for: Early rise in nasal secretory IgA associated with shorter duration of SARS-CoV-2 virus shedding in an acute infection cohort
Source: Front Immunol. 2026 Feb 25;17:1722585. doi: 10.3389/fimmu.2026.1722585 (PMC12975920; doi:10.3389/fimmu.2026.1722585)
Supplement: Supplementary file 4 [file Table1.docx]

**Supplemental Table 1. Analyte/Antigen Naming for Analysis**

| **#** | **Antigen name** | **Analysis Name** | **Description** |
| --- | --- | --- | --- |
| **1** | **2019-nCoV S/293F** | **2P Spike (Wuhan-Hu-1)** | **2-proline stabilized spike trimer** |
| **2** | **229E-RBD** | **229E-RBD** | **Endemic marker** |
| **3** | **HKU1-RBD** | **HKU1-RBD** | **Endemic marker** |
| **4** | **NL63-RBD** | **NL63-RBD** | **Endemic marker** |
| **5** | **OC43-RBD** | **OC43-RBD** | **Endemic marker** |
| **6** | **SARS-CoV-2 DB His** | **RBD** | **Receptor Binding Domain** |
| **7** | **SARS-CoV-2 Nucleoprotein His** | **Nucleoprotein or N-Protein** | **Nucleocapsid Protein** |
| **8** | **SARS-CoV-2 S Hexapro_293F_PPF** | **6P Spike (Wuhan-Hu-1)** | **2-proline stabilized spike trimer** |
| **9** | **SARS-CoV-2 S1 NTD_B.1.1.529_AB** | **B.1.1.529 NTD (Omicron)** | **S1 NTD Omicron Variant** |
| **10** | **SARS-CoV-2 S1 NTD_B.1.1.7_AB** | **B.1.1.7 NTD (Alpha)** | **S1 NTD Alpha Variant** |
| **11** | **SARS-CoV-2 S1 NTD_B.1.351_AB** | **B.1.351 NTD (Beta)** | **S1 NTD Beta Variant** |
| **12** | **SARS-CoV-2 S1 NTD_B.1.617_AB** | **B.1.617 NTD (Delta)** | **S1 NTD Delta Variant** |
| **13** | **SARS-CoV-2 S1 NTD_P.1_AB** | **P.1 NTD (Gamma)** | **S1 NTD Gamma Variant** |
| **14** | **SARS-CoV-2 S_B.1.1.529_AB** | **B.1.1.529 Spike (Omicron)** | **Omicron Spike Trimer** |
| **15** | **SARS-CoV-2 S_B.1.1.7_AB** | **B.1.1.7 Spike (Alpha)** | **Alpha Spike Trimer** |
| **16** | **SARS-CoV-2 S_B.1.351_AB** | **B.1.351 Spike (Beta)** | **Beta Spike Trimer** |
| **17** | **SARS-CoV-2 S_B.1.617_AB** | **B.1.617 Spike (Delta)** | **Delta Spike Trimer** |
| **18** | **SARS-CoV-2 S_P.1_AB** | **P.1 Spike (Gamma)** | **Gamma Spike Trimer** |
| **19** | **SARS-CoV-2-NTD-AVI Biotin** | **NTD (Wuhan-Hu-1)** | **S1 NTD Wuhan-Hu-1** |

**Supplemental Table 2. Median [mix,max] of each shedding duration group for each time point, using data from Figure 1 A.**

|  | | **Shedding Duration Group Median [min, max]** | | |
| --- | --- | --- | --- | --- |
| Isotype | Timepoint | <=7 days | (7, 21] | >21 days |
| SIgA | 1 | 22.43 [0.83, 134.38] | 28.37 [0.12, 561.82] | 52.49 [0.04, 396.39] |
| SIgA | 2 | 23.4 [2.6, 371.98] | 28.06 [0.03, 534.39] | 48.17 [0.39, 380.58] |
| SIgA | 3 | 17.45 [0.82, 364.09] | 37.74 [0.03, 573.72] | 36.29 [0.12, 1079.06] |
| SIgA | 5 | 15.06 [0.11, 245.02] | 32.43 [0.03, 1102.24] | 43.47 [4.14, 299.04] |
| SIgA | 6 | 14.59 [0.16, 135.13] | 22.19 [0.05, 3145.59] | 43.95 [0.44, 275.19] |

**Supplemental Table 3. Geometric Mean Fold Change (Directly related to Figures 1B and 1C data) [calculating the geometric mean first then calculating fold change, truncation was not applied when calculating geometric mean]**

|  | **Isotype Geometric Mean Fold Change (vst 6 vs vst 1)** | |
| --- | --- | --- |
| Analyte | IgA (MFI) | SIgA (SA) |
| HKU1-RBD | 1.00 | 0.96 |
| 229E-RBD | 1.03 | 0.85 |
| NL63-RBD | 1.02 | 0.91 |
| OC43-RBD | 1.04 | 1.03 |
| RBD | 2.90 | 1.47 |
| 2P Spike (Wuhan-Hu-1) | 4.14 | 3.06 |
| 6P Spike (Wuhan-Hu-1) | 5.79 | 4.09 |
| B.1.1.7 Spike (Alpha) | 4.67 | 3.39 |
| B.1.351 Spike (Beta) | 5.97 | 4.11 |
| P.1 Spike (Gamma) | 4.79 | 2.99 |
| B.1.617 Spike (Delta) | 5.41 | 4.48 |
| B.1.1.529 Spike (Omicron) | 4.76 | 2.72 |
| NTD (Wuhan-Hu-1) | 5.12 | 2.29 |
| B.1.1.7 NTD (Alpha) | 2.79 | 1.25 |
| B.1.351 NTD (Beta) | 3.05 | 1.09 |
| P.1 NTD (Gamma) | 3.59 | 1.37 |
| B.1.617 NTD (Delta) | 2.58 | 1.13 |
| B.1.1.529 NTD (Omicron) | 2.73 | 0.97 |
| Nucleoprotein or N-Protein | 2.52 | 1.95 |
